# Photographic Atlas for Cosmetic Noodling Characterization: Development, Analytical Validation, and Consumer Perception

**DOI:** 10.1111/jocd.71244

**Published:** 2026-10-14

**Authors:** Bing Zhang, Lirong Wen, Chenqi Cao, Gabriel Cazorla, Nada Andre, Youcef Ben Khalifa

**Affiliations:** ^1^ Beauty Innovation & Performance Center Chanel China Co. Ltd. Shanghai China; ^2^ Innovation Research & Development Chanel Fragrances & Beauty Pantin France

**Keywords:** consumer acceptance, cosmetic noodling, cosmetic product evaluation, degree of distinguishability, Noodling Atlas

## Abstract

**Background:**

Cosmetic noodling, the formation of particles or strands during product application, negatively impacts user satisfaction and underscores the need for standardized, objective assessment tools.

**Objectives:**

This study aimed to develop, validate, and evaluate consumer acceptance of a standardized Noodling Atlas designed to quantify noodling severity.

**Methods:**

A multiphase approach was employed to create the Noodling Atlas. First, a controlled photographic system was established to generate a comprehensive noodling image library. The Swiss system methodology was then applied for rigorous categorization and refinement of images into seven grades, ranging from Grade 1 (slight noodling) to Grade 7 (severe noodling). For validation, three external experts independently assessed 227 photographs selected from the image library, and intra‐ and interobserver reliability was evaluated using Kappa coefficients and distinguishability indices. Grade 0 (No Noodling) was subsequently added to complete the atlas. Finally, consumer acceptability of noodling grades 0–7 was evaluated through a questionnaire‐based survey administered to a representative sample of Chinese female cosmetic consumers.

**Results:**

Validation demonstrated substantial to almost perfect inter‐ and intraobserver agreement (weighted Kappa coefficients 0.71–0.93), and distinguishability indices ≥ 0.6 confirmed adequate separation between grades. A total of 598 valid questionnaires were collected. Consumer acceptance rates decreased progressively with increasing noodling severity. Notably, acceptance rates did not differ significantly across age groups or city tiers.

**Conclusion:**

The Noodling Atlas is a reliable and valid tool for the objective assessment of noodling severity. Its consistent applicability across demographic subgroups supports its broad utility for cosmetic product development and quality control.

## 1 Introduction

As living standards continue to rise, societal values have shifted markedly toward greater emphasis on personal appearance and skin health. Consequently, high‐quality cosmetics, along with advanced beauty techniques and services, have gained widespread adoption and become integral to daily life [1, 2, 3]. Among these, facial cosmetics, owing to their frequent use and broad appeal, have emerged as one of the most closely scrutinized product categories [4, 5, 6]. While consumers seek tangible benefits such as product efficacy, they are equally attentive to the overall sensory experience, encompassing texture, fragrance, absorption, and post‐application feel [7, 8]. These evolving expectations have prompted ongoing efforts to develop reliable, objective tools for assessing the cosmetic user experience, moving beyond traditional efficacy measures toward more nuanced, user‐centered product evaluation [9, 10, 11, 12].

Cosmetic noodling is a commonly encountered issue in skincare and makeup routines, in which small particles or strands form on the skin's surface during the product application or wear, resembling clumps or flakes [13]. This phenomenon not only hinders the proper product absorption, thereby compromising its effectiveness, but also negatively impacts user satisfaction, which can in turn influence future product selection and usage behavior. Recent studies have identified several contributing factors, including excessive product application, preexisting skin condition, improper layering of products, and incompatibilities between the cosmetic formulation and the skin's characteristics [13, 14].

Despite its widespread occurrence, the assessment of noodling currently relies predominantly on subjective perceptions and experiential judgments from consumers or professionals, which often lack consistency and reproducibility [15]. There is therefore a pressing need for an objective, validated tool capable of accurately quantifying noodling severity. Such a tool would enhance the precision of product evaluations and provide reliable data to support cosmetic research and development, offering actionable insights into product performance and its influence on consumer preferences. In response to this need, the present study aimed to develop a comprehensive Noodling Atlas that quantifies the degree of noodling in facial cosmetics. The atlas underwent rigorous validation by a multidisciplinary panel comprising internal project members with extensive industry experience, dermatologists, and professionals specialized in scale and atlas development. This collaborative process ensured the creation of a practical, valid tool suitable for widespread application. Furthermore, to assess the practical utility of the Noodling Atlas in real‐world contexts, a questionnaire‐based survey was conducted with a representative sample of Chinese cosmetic consumers to evaluate the acceptability of varying degrees of noodling severity.

## 2 Materials and Methods

### 2.1 Study Design

This study employed a two‐phase design encompassing (i) the development and validation of the Noodling Atlas, and (ii) consumer acceptance testing. Following the establishment of noodling grades and expert validation of the final atlas, consumer testing was conducted on the atlas photographs to evaluate how consumers perceive and accept the different grades of noodling.

### 2.2 Development and Validation of the Noodling Atlas

#### 2.2.1 Development of the Noodling Atlas

##### 2.2.1.1 Photo Portfolio Creation

Cosmetic noodling, also referred to as cosmetic pilling [13], is the formation of visible flake‐like, granular, spherical, or noodle‐like aggregates on the skin surface following the application and rubbing of skincare and/or cosmetic products. These aggregates arise from physicochemical interactions among formulation components, product‐to‐product incompatibilities, incomplete product integration, and friction‐induced agglomeration of deposited product materials and desquamated corneocytes. Consequently, cosmetic noodling is distinct from physiological skin scaling and simple residual product accumulation. A structured preparation phase was undertaken to produce a diverse set of images that accurately represent a broad spectrum of noodling severity. A preliminary investigation was first conducted to examine how various cosmetic products and their formulations contribute to the noodling severity. The study focused on products commonly used in skincare routines, including lotions, serums, creams, sunscreens, and primers. These products, either individually or in various combinations, were applied to the face to generate different degrees of noodling. Products selection was based on prior research into the mechanisms underlying noodling formation. To ensure consistency, standardized dosages, adapted to each product texture, were applied evenly across the entire face. This preparation phase aimed to ensure that the resulting images would encompass a wide range of noodling severities, thereby providing a robust foundation for the development of the photographic scales used in subsequent phases of the study.

A controlled photographic system was employed to ensure precise participant positioning and standardized lighting, both critical for achieving consistency and reproducibility in image capture. The participant's face was illuminated using two Godox SK400II flash units, each set to half power and positioned to provide diffused light from fixed angles relative to the camera. This lighting configuration minimized shading and eliminated cast shadows, ensuring uniform illumination across all images. Photography was conducted using a Nikon Z7II camera with a Z 105 mm f/2.8 VR S lens, set to manual mode with an aperture of F/36, exposure time of 1/100 s, ISO 64, and a fixed camera‐to‐subject distance of 50 cm. Images were acquired at a resolution of 8256 × 5504 pixels. The photographic captures were executed either immediately following product application or upon the visual emergence of noodling within an 8‐h period. Color calibration was carried out before each session with a 48‐color patch chart to maintain color accuracy.

The photographic field of view covered key facial areas, including the cheeks, the infraorbital region, part of the ears, and the chin. A trained technician captured high‐resolution digital images, yielding a total of 358 photographs selected for inclusion in the image library. All images were taken with neutral facial expressions (i.e., no smiling, frowning, visible teeth, or partially open eyes), ensuring uniform magnification and consistent image dimensions. The final photo portfolio and Noodling Atlas used in this study were based on images of a 33‐year‐old Chinese female participant with extensive experience using cosmetic products and no dermatologic or systemic conditions that could influence cosmetic performance. The collection comprised 357 photographs representing varying degrees of noodling, alongside a baseline image (Grade 0) showing no visible noodling after product application.

##### 2.2.1.2 Swiss System and Grade Establishment

Four internal project members, with 8–30 years of professional experience in the cosmetics industry, independently selected 100 photographs from a pool of 357 images, aiming to identify those that most accurately represented varying degrees of noodling. Photographs selected by at least two members advanced to the next phase, resulting in a subset of 128 images.

These 128 images were then subjected to the Swiss system, a structured evaluation process involving multiple rounds of pairwise comparisons [16]. In the first round, 64 random pairs were created, and each evaluator selected the image that demonstrated a higher degree of noodling. Following this initial round, the photographs were divided into two subgroups based on their perceived severity. Each subgroup of 64 images underwent another round of pairwise comparisons. This process was repeated over a total of seven rounds, during which images were progressively grouped according to similar levels of noodling severity, ensuring the most precise categorization possible.

After the seven rounds, each image accumulated a score ranging from 0 to 7 per evaluator. The scores from the four project members were summed, yielding a theoretical cumulative score range of 0–28 for each photograph. Based on the actual cumulative scores, the 128 photographs were organized into 25 distinct categories (with empirical score values ranging from 1 to 25). One or two images were selected from each category to best represent the corresponding severity level, yielding a final selection of 28 images. The 28 selected photographs were then reviewed and discussed by the four project members and subsequently grouped into seven categories corresponding to different severity levels of noodling. Within each category, the most representative image was chosen and paired with the baseline photograph (Grade 0) to form the initial version of the Noodling Atlas. A final reevaluation was conducted by the project members to confirm that all photos were accurately assigned to their respective severity grades (Grades 1–7). The specific morphological descriptors and visual criteria defining each severity grade (Grades 0–7) are detailed in Table S1.

#### 2.2.2 Validation of the Noodling Atlas

Three external experts in the cosmetics field conducted two rounds of grading assessments on 227 photos selected from the photo portfolio. Expert 1, a dermatologist with 30 years of professional experience. Expert 2 and Expert 3, both come from professional CROs (Contract research organization) holding certifications from the China Inspection Body and Laboratory Mandatory Approval (CMA) and the China National Accreditation Service for Conformity Assessment (CNAS), with 4 and 9 years of experience in cosmetic efficacy assessment, respectively. Each expert performed two independent rounds of assessments with a minimum interval of 21 days between evaluations.

Given the uneven distribution of noodling severity within the Swiss system, which contained few examples of very low (Grade 1) and very high (Grade 7) noodling severity, all 7 images from these grades were included in the external validation set. For the remaining grades, 110 photographs were randomly selected from the 228 images that had not been part of the Swiss system comparison, and an additional 110 were drawn from the 114 images within the Swiss system (excluding the 7 atlas reference photographs and the 7 Grade 1 and Grade 7 images). To assess grading consistency, both intraobserver reliability (comparing first and second rounds by the same expert) and interobserver reliability (comparing results across experts) were evaluated.

### 2.3 Consumer Testing

Consumers who participated in this phase of the study were recruited from various regions across China between March and April 2024.

#### 2.3.1 Inclusion and Exclusion Criteria

The inclusion criteria were as follows: (1) Chinese nationality, (2) female sex, (3) aged 20–50 years, with an equal distribution between 20–35‐year and 36–50‐year age group (50% each), (4) use of premium skincare brands within the past 12 months; and (5) residence in first‐ or second‐tier Chinese city, with balanced representation across both tiers. The cities selected for participant recruitment included Shanghai, Beijing, Guangzhou, Chengdu, Nanjing, Foshan, Xi'an, Shenyang, Hangzhou, Dongguan, Chongqing, Tianjin, Suzhou, Wuhan, Guiyang, Qingdao, Ningbo, Changsha, Kunming, and Zhengzhou, ensuring coverage of eastern, southern, western, and northern regions of China. All participants provided informed consent prior to inclusion in the study.

#### 2.3.2 Consumer Questionnaire

Consumer acceptance of varying degrees of noodling severity was evaluated through a questionnaire‐based study. Participants were shown individual photographs from the Noodling Atlas displayed on screen, one at a time, and asked to rate whether they found the appearance of each image acceptable or unacceptable. Each participant evaluated a total of eight photographs, corresponding to Grade 0 through Grade 7, to ensure the reliability and consistency of responses.

### 2.4 Statistical Analysis

During the validation process, the order of photograph presentation for scoring was randomized to mitigate potential bias. Reproducibility of the Noodling Atlas grading was assessed using two complementary statistical methods. First, intraobserver agreement (consistency of the same observer across repeated assessments) and interobserver repeatability (consistency across different observers) were quantified using the weighted Kappa coefficient [17]. Interpretation of Kappa values followed the guidelines of Landis and Koch: values < 0.2 indicated slight agreement, 0.21–0.40 fair agreement, 0.41–0.60 moderate agreement, 0.61–0.80 substantial agreement, and 0.81–1.00 almost perfect agreement [18]. Second, the degree of distinguishability (DD) between adjacent categories of the grading scale was assessed using a log‐linear nonuniform association model [19]. DD values range from 0 (not distinguishable) to 1 (perfectly distinguishable), with values ≥ 0.6 indicating good distinguishability between grades. In cases where multiple observers had difficulty distinguishing between two adjacent grades, the grade in question was either removed from the scale or its representative photograph replaced to improve clarity. DD estimates were reported along with their 95% confidence intervals (CIs), where applicable. Additionally, to explore potential demographic variations in consumer acceptance, subgroup analyses based on age groups (20–35 and 36–50 years) and city tiers (Tier 1 and Tier 2) were performed using Pearson's *Chi‐square* test or *Fisher's* exact test. A two‐sided *p*‐value < 0.05 was considered statistically significant. All statistical analyses were performed using R software.

## 3 Results

### 3.1 Development of the Noodling Atlas

#### 3.1.1 Swiss System

Following the seven rounds of the Swiss system, the 128 photographs were categorized into 25 distinct groups based on their cumulative competition scores. The distribution of photographs across these groups is summarized in Table 1.

**TABLE 1:** Swiss system.

Scores	Number of photos (*N* = 128)
1	2
2	1
3	1
4	2
5	4
6	4
7	9
8	2
9	4
10	7
11	8
12	8
13	7
14	8
15	11
16	9
17	8
18	5
19	6
20	4
21	9
22	3
23	3
24	1
25	2

#### 3.1.2 Noodling Atlas

The final Noodling Atlas developed in this study consists of eight photographs representing varying degrees of noodling severity, from Grade 0 (no visible noodling) to Grade 7 (most severe noodling) (Figure 1). Mild severity (Grades 1–2) is primarily characterized by fine aggregates. As severity increases, these aggregates evolve into distinct flake‐like and granular forms (Grades 3–5). At the most severe stages (Grades 6–7), large, elongated noodle‐like aggregates become predominant and are distributed extensively across the evaluated area.

**FIGURE 1:**
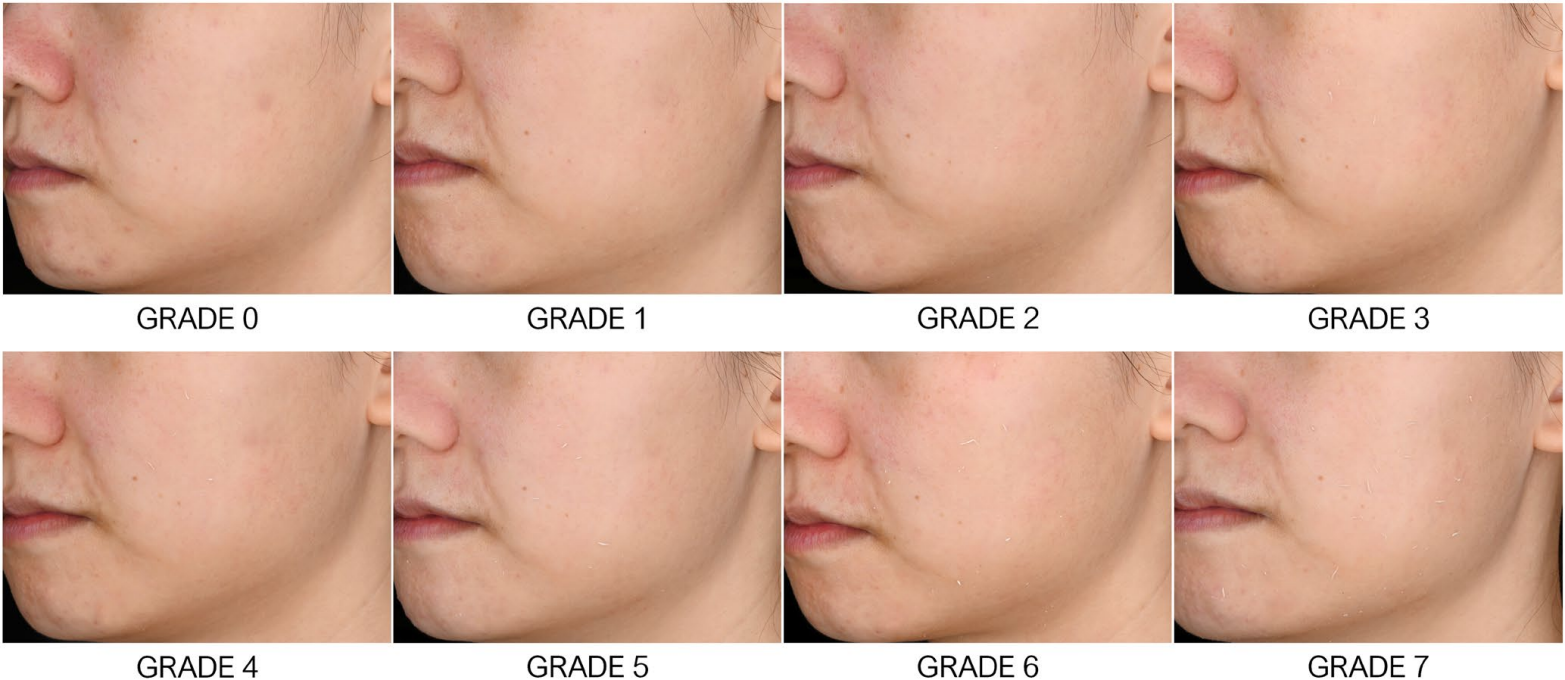
The Noodling Atlas.

### 3.2 External Validation of the Noodling Atlas

Interobserver agreement and intraobserver reproducibility were assessed based on the Noodling Atlas grading criteria. Agreement among the three experts across two rounds of evaluation on the 227 selected photographs ranged from substantial to almost perfect, with weighted Kappa coefficients between 0.71 and 0.93 (Table 2).

**TABLE 2:** Intraobserver and interobserver reliability.

Weighted quadratic kappa coefficient	Expert 1	Expert 2	Expert 3
Expert 1	0.82	0.84 (first round)	0.73 (first round)
Expert 2	0.81 (second round)	0.93	0.74 (first round)
Expert 3	0.78 (second round)	0.71 (second round)	0.91

*Note:* Kappa coefficient according to Landis and Koch [18]: < 0.2 represents slight agreement; 0.21–0.40 fair agreement; 0.41–0.60 moderate agreement; 0.61–0.80 substantial agreement; and 0.81–0.90 strong agreement; 0.91–1.00 almost perfect agreement.

The degree of distinguishability (DD) between adjacent grades was evaluated for both intra‐ and interobserver assessments and demonstrated good separation, with DD values exceeding 0.6 for most comparisons. The only exception was between Grade 4 and Grade 5 during the second round of evaluations by two of the three raters (Figure 2). Overall, these results indicate that the grades in the Noodling Atlas are well‐distinguished, and no revisions to the scale were deemed necessary.

**FIGURE 2:**
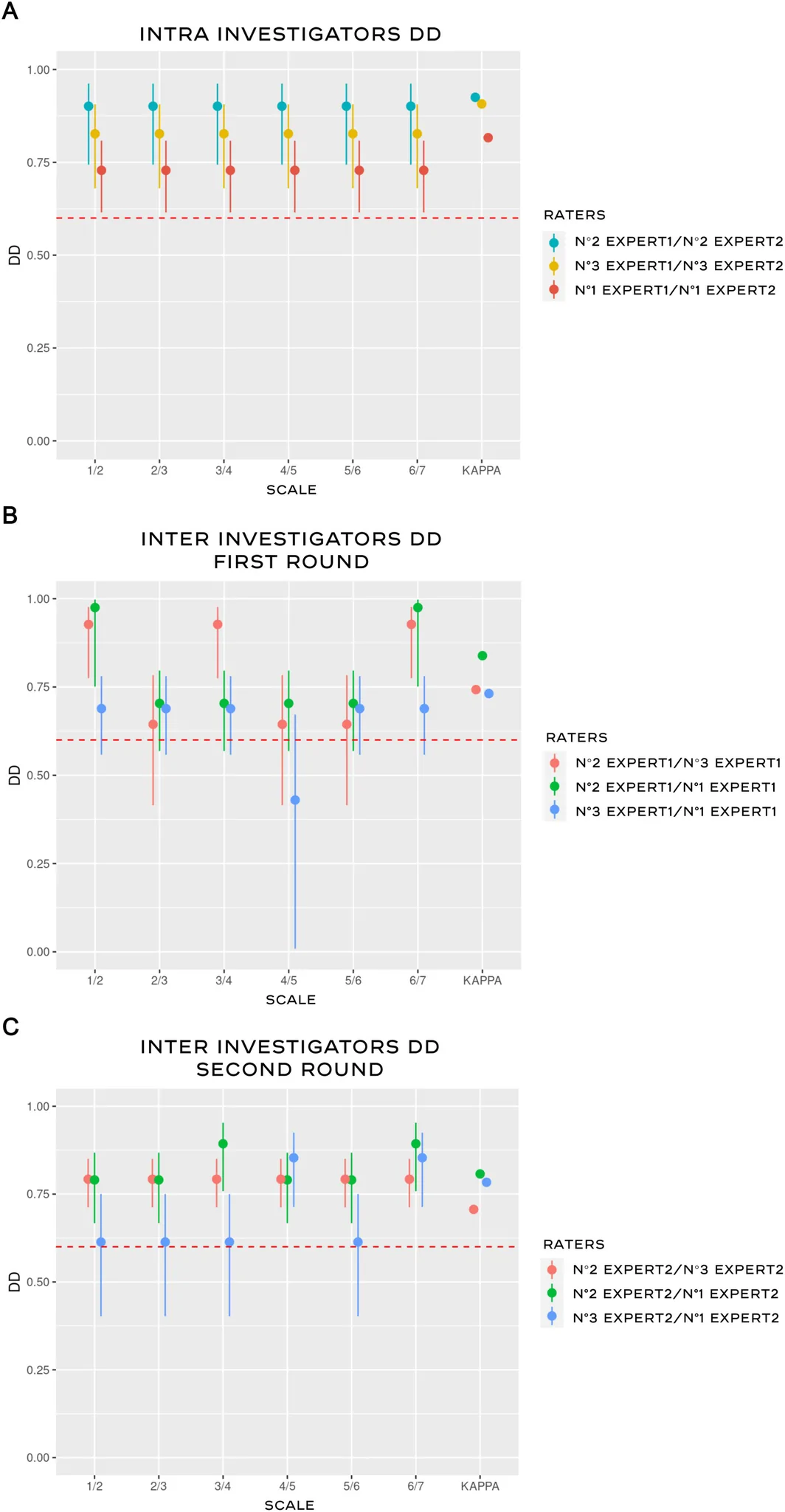
Degree of distinguishability (DD) between adjacent categories in the external validation of Noodling Atlas. The DD values range from 0 (not distinguishable) to 1 (perfectly distinguishable), with DD values ≥ 0.6 indicating good distinguishability between grades. (A) Intraobserver DD; (B) Interobserver DD in the first round; and (C) Interobserver DD in the second round.

### 3.3 Consumer Testing

#### 3.3.1 Consumer Characteristics

A total of 608 female cosmetic consumers from first‐ and second‐tier cities in China completed the questionnaire survey, of whom 598 provided valid responses. The mean age of the participants was 32.25 ± 7.12 years, with 340 (56.86%) in the 20–35 age group and 258 (43.13%) in the 36–50 age group. Geographically, participants were distributed across four regions in China: 146 (24.46%) from the eastern region, 143 (23.95%) from the southern region, 155 (25.96%) from the western region, and 154 (25.63%) from the northern region. Of the total, 282 (47.16%) resided in first‐tier cities, while 316 (52.84%) resided in second‐tier cities (Table 3).

**TABLE 3:** Consumer characteristics.

Variables	Consumer (*N* = 598)
Age (years), mean ± SD	32.25 ± 7.12
Age group (years), *n* (%)	
20–35	340 (56.86)
36–50	258 (43.14)
Female, *n* (%)	598 (100)
Residential city, *n* (%)	
Tier 1 cities	282 (47.16)
Tier 2 cities	316 (52.84)
Residential regions, *n* (%)	
East	146 (24.41)
South	143 (23.91)
West	155 (25.92)
North	154 (25.75)

Abbreviation: SD, standard deviation.

#### 3.3.2 Consumer Questionnaire

The questionnaire assessed consumer acceptability of noodling severity levels as represented in the Noodling Atlas. All 598 participants (100%) rated Grade 0 (no noodling) as acceptable. Acceptability rates declined progressively with increasing noodling severity: 587 (98.16%) found Grade 1 acceptable, 579 (96.82%) found Grade 2, 471 (78.76%) found Grade 3, 368 (61.54%) found Grade 4, 188 (31.44%) found Grade 5, 49 (8.19%) found Grade 6, and 26 (4.35%) found Grade 7 acceptable (Table 4). A marked decrease in acceptability was observed between Grades 2 and 3, where the acceptance rate dropped by approximately 18 percentage points.

**TABLE 4:** Consumer questionnaire acceptance.

Photo grades, *n* (%)	Consumer (*N* = 598)
Grade 0	598 (100.00)
Grade 1	587 (98.16)
Grade 2	579 (96.82)
Grade 3	471 (78.76)
Grade 4	368 (61.54)
Grade 5	188 (31.44)
Grade 6	49 (8.19)
Grade 7	26 (4.35)

#### 3.3.3 Subgroup Analysis of Consumer Acceptance

Subgroup analyses were conducted to compare consumer acceptance rates across demographic variables, specifically age groups and city tiers (Table S2).

At Grade 3, the acceptance rates were 80.29% for the 20–35 age group and 76.74% for the 36–50 age group, with no statistically significant difference (*p* = 0.293). Similarly, at Grade 4, the rates were 60.29% and 63.18%, respectively (*p* = 0.473). At Grade 5, acceptance rates were 32.06% for the younger group versus 30.62% for the older group (*p* = 0.707). Regarding cities tier, acceptance rates at Grade 3 were 78.72% in Tier 1 cities and 78.80% in Tier 2 cities (*p* = 0.982), and at Grade 5, 31.56% for Tier 1 cities versus 31.33% for Tier 2 cities (*p* = 0.952). Overall, no statistically significant differences were observed between the analyzed demographic subgroups at any noodling severity grade (all *p* > 0.05).

## 4 Discussion

This study successfully developed and validated the Noodling Atlas, an objective tool for assessing varying degrees of facial cosmetic noodling. The multistage development process ensured a reliable representation of noodling severity and external validation confirmed strong inter‐ and intraobserver consistency as well as clear distinguishability between grades. The consumer survey further demonstrated the practical relevance of the Noodling Atlas, revealing a progressive decline in acceptance as noodling severity increased. Collectively, these findings underscore the utility of the Noodling Atlas as a valid and objective assessment tool, offering actionable insights for both cosmetic product development and the understanding of consumer preferences.

Cosmetic noodling is a prevalent issue, yet systematic research into its causes and standardized methods of assessment remain limited [13]. In recent years, visual atlases have emerged as effective tools for establishing standardized references in dermatology and cosmetic science. Such atlases, for instance those used for skin aging or tone classification, enable precise grading of age‐related or pigmentation changes using standardized photographic scales [20, 21]. The development of the Noodling Atlas aligns with these advancements, providing a robust, reproducible, and objective approach to quantifying noodling severity. Moreover, the integration of atlas‐based tools into cosmetic evaluation processes builds on advances in photographic and surface analysis techniques, which are now fundamental to assessing the efficacy and safety of cosmetic products [22]. By establishing a standardized framework for evaluating noodling, this study contributes to the broader goals of cosmetic research, supporting both the refinement of product formulations and an improved understanding of their impact on consumer satisfaction.

Through a multistage process, the atlas was developed and refined to accurately represent the full spectrum of noodling severity. Initial screening excluded images that lacked typical noodling characteristics, ensuring the selection of relevant photographs. Subsequent rounds incorporated the Swiss system methodology, a tournament‐style ranking process that enabled precise categorization of noodling severity through multiple independent evaluations [16]. This approach minimized subjective bias and ensured representative grading by leveraging assessments from four experienced judges. The iterative nature of the process allowed for the systematic refinement, culminating in a comprehensive atlas. The final Noodling Atlas comprises eight photographs representing grades ranging from no visible noodling (Grade 0) to the most severe noodling observed (Grade 7) with each grade systematically categorized to ensure clarity and distinctiveness. The Swiss system, originally devised for competitive ranking in chess tournaments [23], proved highly effective in this context. Its application facilitated equitable and objective scoring across multiple rounds, ensuring that each image was evaluated thoroughly and consistently. This study demonstrates the utility of structured, multiphase methodologies such as the Swiss system in mitigating subjective variability in cosmetic evaluation, setting a new standard for reproducibility and precision in this field.

External validation is indispensable in the health sciences, ensuring that newly developed assessment tools, standards, and research outcomes are both reliable and generalizable. Its importance is well‐documented across multiple domains. In the development of measurement instruments, validation processes confirm that tools accurately assess intended constructs and produce consistent results across diverse populations and settings [24]. In the establishment of standards, validation practices ensure that benchmarks are applicable and effective in varied contexts, thereby enhancing their credibility and utility [25]. In scientific research, particularly in the evaluation of clinical prediction models, external validation is critical for assessing predictive performance across independent datasets, ensuring relevance and effectiveness in real‐world applications [26]. The present findings align with these principles. Independent external validation demonstrated strong concordance in photographic ratings and robust reproducibility in distinguishing closely related and potentially ambiguous grades, highlighting the methodological rigor of the atlas's grading system. Each category was designed to represent distinct characteristics, thereby minimizing overlap and ambiguity.

Notably, the Noodling Atlas demonstrates broad generalizability across diverse consumer demographics. Subgroup analyses revealed no statistically significant differences in the acceptability of varying noodling severities across age cohorts (20–35 vs. 36–50 years) or geographic strata (Tier 1 vs. Tier 2 cities). This suggests that the perception of, and tolerance for, cosmetic noodling are largely uniform among Chinese female consumers. Previous research on cosmetic sensory evaluation indicates that while specific formulation preferences—such as texture, hydration, and fragrance—may vary according to demographic factors including age or geographic location [6], aversion to fundamental application defects, such as pilling or noodling, tends to be universally shared [13, 15]. These consistent subgroup findings reinforce the position of the Noodling Atlas as a robust, unbiased tool for cosmetic evaluation and quality control.

The development of any evaluation tool for cosmetic efficacy or user experience must address consumer needs and accurately reflect their preferences. The Noodling Atlas fulfills these requirements, as demonstrated by a representative survey of Chinese consumers. The results revealed a marked decline in consumer acceptance with increasing noodling severity, from 100% at Grade 0 to only 4.35% at Grade 7, underscoring the substantial impact of noodling on user experience and, by extension, on purchase intentions. By providing an objective and standardized method for assessing noodling severity, the atlas offers considerable practical value. It establishes a scientific basis for cosmetic development, enabling manufacturers to address noodling proactively and optimize product formulations. Furthermore, the atlas has potential applications in product quality control, helping to ensure consistency across batches and product combinations and to maintain noodling levels that are acceptable to consumers. These findings highlight the importance of integrating consumer‐centric perspectives into the development of cosmetic evaluation tools. The visual appearance of cosmetic noodling may vary depending on individual skin surface characteristics, lighting conditions, and imaging methodologies. Consequently, the photographic atlas was developed using a single subject and standardized imaging parameters to control these background variables and maintain visual consistency. A resulting limitation of this study is the lack of diverse Fitzpatrick skin types. Future research is required to validate this assessment framework across multi‐skin tone populations.

## 5 Conclusion

This study developed and validated a standardized objective tool, the Noodling Atlas, for evaluating the severity of noodling in facial cosmetics. The atlas provides a reliable and reproducible framework for assessing noodling severity, addressing a previously unmet need in cosmetic evaluation. By establishing standardized grading criteria with demonstrated cross‐demographic applicability, the Noodling Atlas offers a scientific basis for cosmetic manufacturers and professionals to optimize product formulations and enhance user satisfaction. Future studies should explore the applicability of the atlas across additional demographic groups, skin types, and product categories, as well as its integration into routine quality control processes.

## Author Contributions

All authors jointly participated in the entire project, including study design, photo shooting, evaluation, etc. They made equal contributions to this article.

## Ethics Statement

The authors have nothing to report.

## Conflicts of Interest

The authors declare no conflicts of interest.

## Supporting information


**Table S1:** Morphological descriptors and visual detectability criteria for cosmetic noodling severity grades.
**Table S2:** Subgroup analysis of consumer acceptance across varying noodling grades stratified by age and city tier.

## Data Availability

The data that supports the findings of this study are available in the Supporting Information of this article.
