# Supplementary material for: Photographic Atlas for Cosmetic Noodling Characterization: Development, Analytical Validation, and Consumer Perception
Source: J Cosmet Dermatol. 2026 Oct 14;25(10):e71244. doi: 10.1111/jocd.71244 (PMC13669516; doi:10.1111/jocd.71244)
Supplement: Supplementary file 1 [file JOCD-25-e71244-s001.docx]

**Supplementary materials**

Supplementary Table S1. Morphological descriptors and visual detectability criteria for cosmetic noodling severity grades.

| **Grade** | **Severity Category** | **Morphological Descriptors and Visual Detectability Criteria** |
| --- | --- | --- |
| **0** | No visible noodling | Complete absence of visually detectable aggregates on the skin surface. |
| **1** | Barely detectable | Presence of extremely sparse, fine flake-like or granular aggregates that are scarcely perceptible under normal viewing conditions. |
| **2** | Detectable | Presence of sparse, small flake-like, granular, or spherical aggregates that can be identified only upon careful, close-range inspection. |
| **3** | Noticeable | Presence of distinct flake-like, granular, or spherical aggregates that are observable at close viewing distance but remain limited in number and distribution. |
| **4** | Clearly visible | Presence of readily visible granular, spherical, or short noodle-like aggregates that contrast with the surrounding skin surface. |
| **5** | Obvious | Presence of numerous granular, spherical, and noodle-like aggregates with increased size and distribution, visibly compromising the uniform appearance of the skin surface. |
| **6** | Pronounced | Extensive accumulation of prominent noodle-like aggregates and clustered particulate aggregates  that are highly conspicuous without intentional inspection. |
| **7** | Extensive | Widespread distribution of dense, large, and highly visible aggregates, resulting in severe disruption of the cosmetic finish across the application area. |

Supplementary Table S2. Subgroup analysis of consumer acceptance across varying noodling grades stratified by age and city tier.

| Grade | Age group | | | Residential city | | |
| --- | --- | --- | --- | --- | --- | --- |
|  | 20-35 years | 36-50 years | P | Tier 1 | Tier 2 | P |
| 0, n(%) | 340 (100) | 258 (100) | - | 282 (100) | 316 (100) | - |
| 1, n(%) | 334 (98.24) | 253 (98.06) | 0.876 | 276 (97.87) | 311 (98.42) | 0.620 |
| 2, n(%) | 326 (95.88) | 253 (98.06) | 0.132 | 273 (96.81) | 306 (96.84) | 0.985 |
| 3, n(%) | 273 (80.29) | 198 (76.74) | 0.293 | 222 (78.72) | 249 (78.80) | 0.982 |
| 4, n(%) | 205 (60.29) | 163 (63.18) | 0.473 | 177 (62.77) | 191 (60.44) | 0.560 |
| 5, n(%) | 109 (32.06) | 79 (30.62) | 0.707 | 89 (31.56) | 99 (31.33) | 0.952 |
| 6, n(%) | 27 (7.94) | 22 (8.53) | 0.796 | 25 (8.87) | 24 (7.59) | 0.572 |
| 7, n(%) | 18 (5.29) | 8 (3.10) | 0.193 | 11 (3.90) | 15 (4.75) | 0.613 |
